# Anti-Proliferation Effect of Theasaponin E_1_ on the ALDH-Positive Ovarian Cancer Stem-Like Cells

**DOI:** 10.3390/molecules23061469

**Published:** 2018-06-17

**Authors:** Ling-Yan Jia, Hui-Long Xia, Zhi-Da Chen, Casey Compton, Heather Bucur, Devendra A. Sawant, Gary O. Rankin, Bo Li, You-Ying Tu, Yi Charlie Chen

**Affiliations:** 1 Department of Tea Science, Zhejiang University, Hangzhou 310058, China; 11416013@zju.edu.cn (L.-Y.J.); 21616101@zju.edu.cn (Z.-D.C.); drlib@zju.edu.cn (B.L.); 2 College of Science, Technology and Mathematics, Alderson Broaddus University, Philippi, WV 26416, USA; ccompton@k12.wv.us (C.C.); bucurhm@battlers.ab.edu (H.B.); 3 School of Environmental Science and Engineering, Zhejiang Gongshang University, Hangzhou 310018, China; hlxia@zjgsu.edu.cn; 4 School of Anatomical Science, Alderson Broaddus University, College of Medical Science, Philippi, WV 26416, USA; drdsawant@gmail.com; 5 Department of Pharmacology, Physiology and Toxicology, Joan C. Edwards School of Medicine, Marshall University, Huntington, WV 25755, USA; rankin@marshall.edu

**Keywords:** theasaponin E_1_, *Camellia sinensis*, cancer stem cells, ALDH, ovarian cancer

## Abstract

Ovarian cancer has the highest mortality rate of all gynecological malignancies and the five-year death rate of patients has remained high in the past five decades. Recently, with the rise of cancer stem cells (CSCs) theory, an increasing amount of research has suggested that CSCs give rise to tumor recurrence and metastasis. Theasaponin E_1_ (TSE_1_), which was isolated from green tea (*Camellia sinensis*) seeds, has been proposed to be an effective compound for tumor treatment. However, studies on whether TSE_1_ takes effect through CSCs have rarely been reported. In this paper, ALDH-positive (ALDH+) ovarian cancer stem-like cells from two platinum-resistant ovarian cancer cell lines A2780/CP70 and OVCAR-3 were used to study the anti-proliferation effect of TSE_1_ on CSCs. The ALDH+ cells showed significantly stronger sphere forming vitality and stronger cell migration capability. In addition, the stemness marker proteins CD44, Oct-4, Nanog, as well as Bcl-2 and MMP-9 expression levels of ALDH+ cells were upregulated compared with the original tumor cells, indicating that they have certain stem cell characteristics. At the same time, the results showed that TSE_1_ could inhibit cell proliferation and suspension sphere formation in ALDH+ cells. Our data suggests that TSE_1_ as a natural compound has the potential to reduce human ovarian cancer mortality. However, more research is still needed to find out the molecular mechanism of TSE_1_-mediated inhibition of ALDH+ cells and possible drug applications on the disease.

## 1. Introduction

Ovarian cancer has the highest mortality rate of all gynecological malignancies [1], resulting in approximately 22,280 new cases being diagnosed and 14,240 deaths occuring every year in the United States [2]. Around 70% patients fall into relapse and experience chemo-resistance, and the five-year survival rate remains less than 40% [3]. Recently, with the rise of cancer stem cell (CSCs) theory, increasingly more research results have shown that CSCs have typical stem cell characteristics, such as self-renewal, multidifferentiation, and tumorigenicity etc., [4]. CSCs have been confirmed and isolated in various solid tumors, such as breast cancer [5], ovarian cancer [6], pancreas cancer [7], colon cancer [8] and acute myeloid leukemia [9]. Meanwhile, increasingly more studies have suggested that CSCs contribute to tumor growth, metastasis, chemo-resistance and recurrence with the neoplasm renewal source. In addition, the existence of CSCs is also a key reason for the failure of conventional tumor treatments [10]. Thus far, the biomakers including CD44, CD133, CD117, CD24 and aldehyde dehydrogenase (ALDH) have been extensively studied to differentiate and identify CSCs [1,6]. Among these, studies suggest that ALDH may be involved in the epithelial-mesenchymal transition of tumor cells, allowing cancer cells to evade T-cell-mediated cytolysis, and contributing to the oxidation of retinol to retinoic acid in early stem cell differentiation [11]. At present, ALDH+ cells exhibited tumorigenic and chemo-resistance capacity, and proved to be associated with poor patient outcome in high-grade serous ovarian cancer [6].

Selectively targeting CSCs is a new strategy for tumor treatment. At the same time, natural compounds have received increasing attention in the research of cancer and CSCs due to their high activity and low number of side effects. The saponins from Rhizoma paridis have shown the inhibitory effect on growth of CSCs in non-small cell lung cancer [12]. EGCG and TFs can inhibit the self-renewal capacity of prostate CSCs, in part by downregulating Bmi1 gene expression [13]. Theasaponin E_1_ (TSE_1_), an acylated oleanene-type triterpene ologoglycoside with the molecular formula C_59_H_90_O_27_, was firstly isolated from green tea [*Camellia sinensis* (L.) O. Kuntze] seeds in 1998 [14]. This compound has been proven to possess various activities. TSE_1_ could alleviate the salt tolerance of yeasts [15], and inhibit ethanol-induced gastric mucosal lesions [16], angiogenesis and obesity [17]. It could also suppress the human tumor cell lines K562 and HL60 [18]. Our previous study showed that the total saponins from *C. sinensis* seeds exhibit potent growth inhibitory effect on two cisplatin-resistant ovarian cancer cell lines A2780/CP70 and OVCAR-3 [19]. 

In this study, A2780/CP70 and OVCAR-3 cell lines were chosen. The tumor sphere culture method was selected to accumulate CSCs. ALDH was used as a stem cell surface marker to indirectly detect the proportion of CSCs. TSE_1_ was selected as the experimental drug. The purpose is to detect the population of ALDH+ cells that were accumulated in two ovarian cancer cell lines and determine if those cells have certain stem cell characteristics, then investigate the effect of TSE_1_ on the ALDH+ cells.

## 2. Results

### 2.1. Expression of ALDH in Both Tumor and Sphere Cells

**Figure 1:**
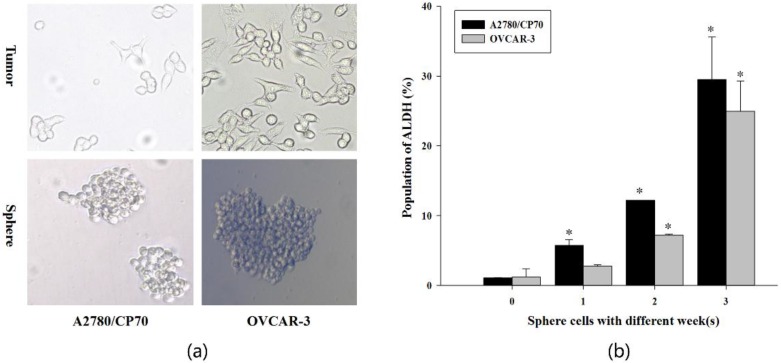
The population of ALDH of both tumor and sphere cells cultured in serum-free medium with different weeks from A2780/CP70 and OVCAR-3 cell lines. (**a**) morphological photographs of ovarian tumor and sphere cells (3-weeks) for both two cell lines (200×); (**b**) ALDH ratio after culturing in serum-free medium could accumulate in a time-dependent manner. Data was expressed as percent of ALDH+ cells and shown as mean ± SD (*n* = 3), * = *p* ≤ 0.05, a significant difference compared with zero-time control.

According to the basic principle of serum-free culture, the differentiated mature tumor cells cannot adhere to the wall and go to apoptosis in the serum-free state, whereas undifferentiated CSCs within total tumor cells can grow and experience multidifferentiation into both tumor and CSCs to form a spherical aggregate state, thus differentiating from each other. The spheres derived from A2780/CP70 and OVCAR-3 cells appeared and completely formed within seven days (one week). It can be seen in Figure 1a that two ovarian cancer cells exhibit spindle or oval-shaped single cell distribution in the adherent culture, but, in the serum-free culture state, both cells showed different degrees of spherical dense multicellular aggregation and were able to float in the culture fluid. The results indicated the presence of CSCs in both ovarian cancer cells. An ALDEFLUOR Stem Cell Identification Kit was used to examine the proportion of ALDH+ cells in the tumor and different culture algebraic suspension cells. The experimental results showed (Figure 1b) that ALDH+ cells ratio was 1.05%, 5.75%, 12.20% and 29.50% in tumor cells and sphere cells with 1-week, 2-weeks and 3-weeks in A2780/CP70, and 1.25%, 2.75%, 7.20%, 24.95% in OVCAR-3, respectively. Our results indicated that there was indeed a very small amount of ALDH+ cells in both ovarian cancer cells, which was less than 2.0%, while the proportion of ALDH+ cells in two cell lines showed a significant increase trend in a time-dependent manner. In addition, the increasing trend of the A2780/CP70 cell line and the proportion of ALDH+ cells were higher than that of OVCAR-3. The proportion of ALDH+ cells in both of the suspension spheres after three generations of culture exceeded 20%, indicating that the serum-free suspension culture method can significantly enrich CSCs, and is a simple and effective enrichment method. 

### 2.2. Sphere Cells Exhibits Stemness Properties

**Figure 2:**
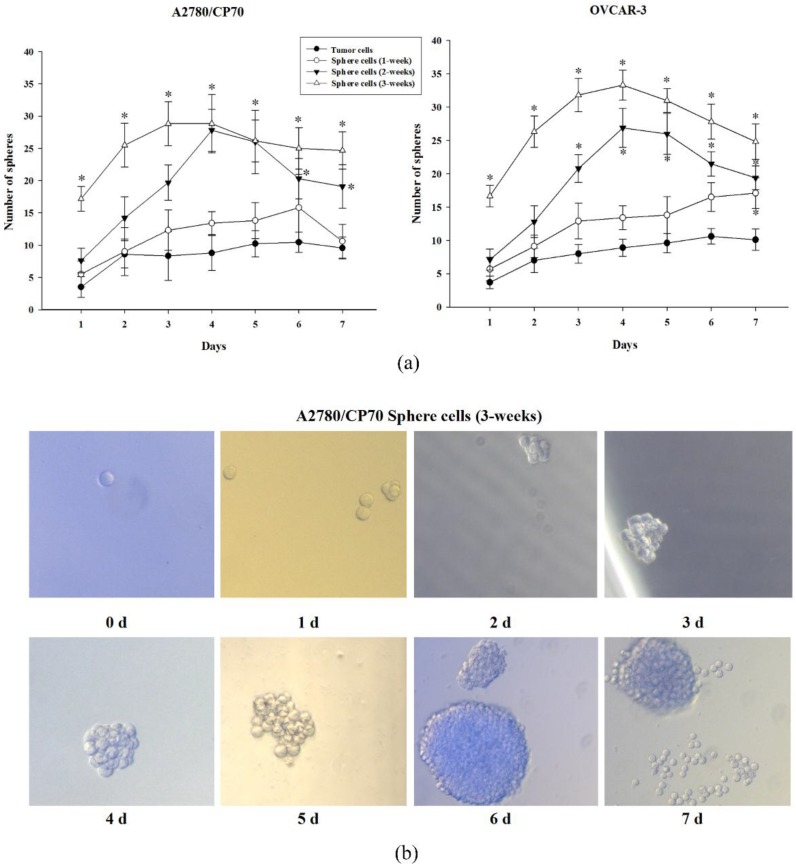
Sphere cells from A2780/CP70 and OVCAR-3 cell lines possess the stemness properties. (**a**) both A2780/CP70 and OVCAR-3 sphere cells had higher capacities in sphere formation compared to tumor cells; (**b**) the spheroidal morphology observed under a light microscope after culturing in serum-free medium (A2780/CP70 sphere cells 3-weeks) (200×). Data was expressed as number of spheres and shown as mean ± SD (*n* = 6), * = *p* ≤ 0.05, significant difference compared with tumor cells at the same time.

The single cell sphere formation ability experimental results showed (Figure 2a) that the average number of suspended spheres after one week culturing of tumor cells (0 generation) was only about 10. From the first generation to the third generation of suspension cells, the average number of suspended spheres increased significantly after one week of culture, indicating that the proportion of ALDH+ cells was positively correlated with the single cell pelleting ability. Observing the number of spheres of different generations of cells in different cultures on the same day, the same rule was found, and, in particular, the ability of the third generation cells was significantly increased. It was confirmed that ALDH+ cells have stronger single-cell spherule ability, and this ability increases as the proportion of ALDH+ cells increases. Interestingly, the second-generation, third-generation cells showed a trend of firstly increased and then a stable or slightly decreased number of spheres, which is different from the trend of zero generation in the control group. Furthermore, we used A2780/CP70 cells as an example to observe the state of cell growth and the state of sphere formation daily under a microscope (Figure 2b). Day 0 cells presented a single cell suspension state. From the first day, it can be observed that some cells show a globular aggregation, two or three or four cells in a cluster or linear or spherical state states can be found in the field of vision; the second, third and fourth days were the period of rapid spheres formation, and aggregated spherical cell clusters of different sizes appeared in the visual field. From the fifth day to the seventh day, contact between adjacent cell masses forming a larger cell mass can only be included once in the count, which was probably the reason for the decrease in the number of sphere cells. Another interesting phenomenon was that, on the sixth or seventh day, we observed that the cell cluster appeared with black shadows, the cell contours became blurred, and a single cell “escape” phenomenon appeared at the edge of the spherical cells. We inferred that it may be caused by the migration of cells to a more favorable environment in the harsh environment of dense spherical cells in the absence of oxygen and nutrient delivery. To confirm whether ALDH+ cells have a stronger ability to metastasize and invade, we conducted Transwell chamber experiments and screened ALDH+ cells (three generation) as follow-up study subjects. The above results demonstrated that ALDH+ cells, especially third-generation cells, have stronger capabilities than tumor mother cells, which can be used as a manifestation of the characteristics of cancer stem cells.

**Figure 3:**
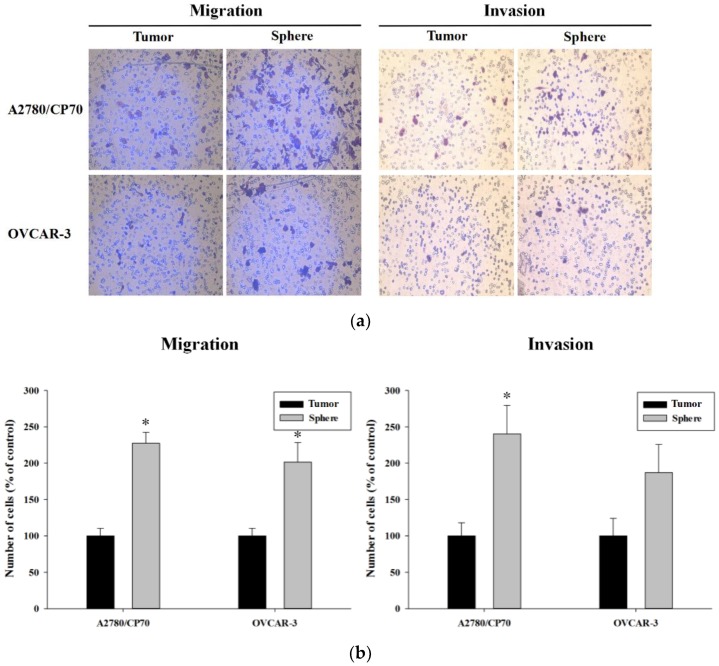
Sphere cells (3-weeks) from A2780/CP70 and OVCAR-3 cell lines possess the stemness properties. (**a**) the number of cells through the membrane was observed under a light microscope after Transwell for migration and invasion assays (100×); (**b**) both A2780/CP70 and OVCAR-3 sphere cells had significantly enhanced migration ability, and higher invasion viability only for A2780/CP70 cell line but not OVCAR-3. Data was expressed as number of spheres and shown as mean ± SD (*n* = 5), * = *p* ≤ 0.05, significant difference compared with tumor cells.

Furthermore, the results of the Transwell chamber showed that, in terms of cell migration ability, both ALDH+ cells exhibited a significantly higher migration potential (Figure 3a). Invasion experiments showed that ALDH+ cells did not significantly change the invasive ability for OVCAR-3 cell line, while ALDH+ cells in A2780/CP70 cell line have significantly increased invasive ability. The above results demonstrated that ALDH+ cells were more potent than tumor cells in terms of migration, and can serve as a manifestation of the biological characteristics of cancer stem cells.

**Figure 4:**
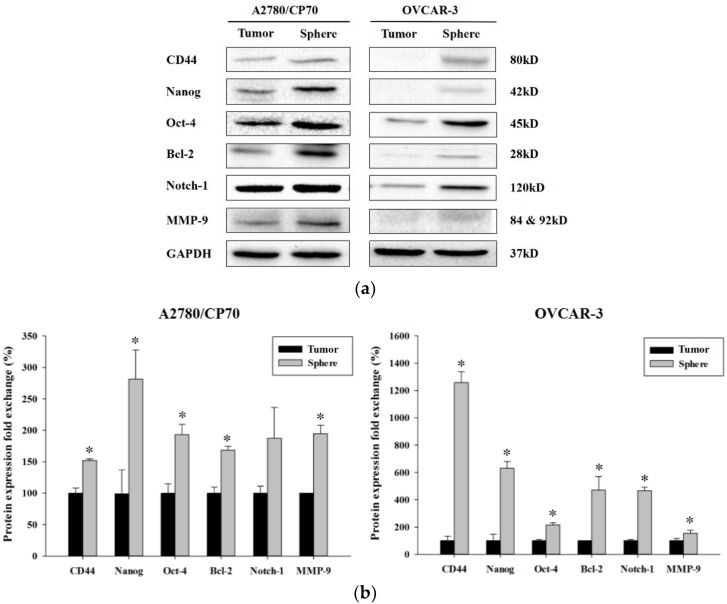
Sphere cells (3-weeks) from A2780/CP70 and OVCAR-3 cell lines possess the stemness properties. (**a**) the results of protein strips of CD44, Nanog, Oct-4, Bcl-2, Notch-1 and MMP-9 proteins; (**b**) both A2780/CP70 and OVCAR-3 sphere cells had marked higher expression of CD44, Nanog, Oct-4, Bcl-2 and MMP-9 proteins compared with tumor cells. Data was expressed as the number of spheres and shown as mean ± SD (*n* = 3), * = *p* ≤ 0.05.

Western blot analysis showed in Figure 4, the expression levels of stemness marker proteins CD44, Nanog, and Oct-4 in ALDH+ cells were significantly higher than those in tumor cells for both cell lines. In addition, the expression level of key proteins found to be over-expressed frequently existed in cancer stem cells, and anti-apoptotic protein Bcl-2, Notch-1 and MMP-9 were detected to increase in different degrees. Bcl-2 protein was significantly increased in both cells, Notch-1 protein was significantly changed in OVCAR-3 cells, but not in A2780/CP70 cells. Observing the expression of MMP-9 protein, both ALDH+ cells were significantly increased, which was consistent with the result of the cell migration ability detected by Transwell chamber assay. At the same time, we compared the A2780/CP70 and OVCAR-3 cells, both in tumor and ALDH+ cells, finding that the expression level of MMP-9 protein was higher in A2780/CP70 than OVCAR-3 cells. This result was also consistent with Transwell’s results. The above results indicated that ALDH+ cells exhibit certain stem cell biological characteristics from the molecular level.

Taken together, our findings indicated that ALDH+ cells (3-weeks) accumulated with serum-free culture have certain biological characteristics of CSCs, and can be considered as ovarian cancer stem-like cells for follow-up experiments. 

### 2.3. TSE_1_ Inhibits the Cell Growth of Sphere Cells

**Figure 5:**
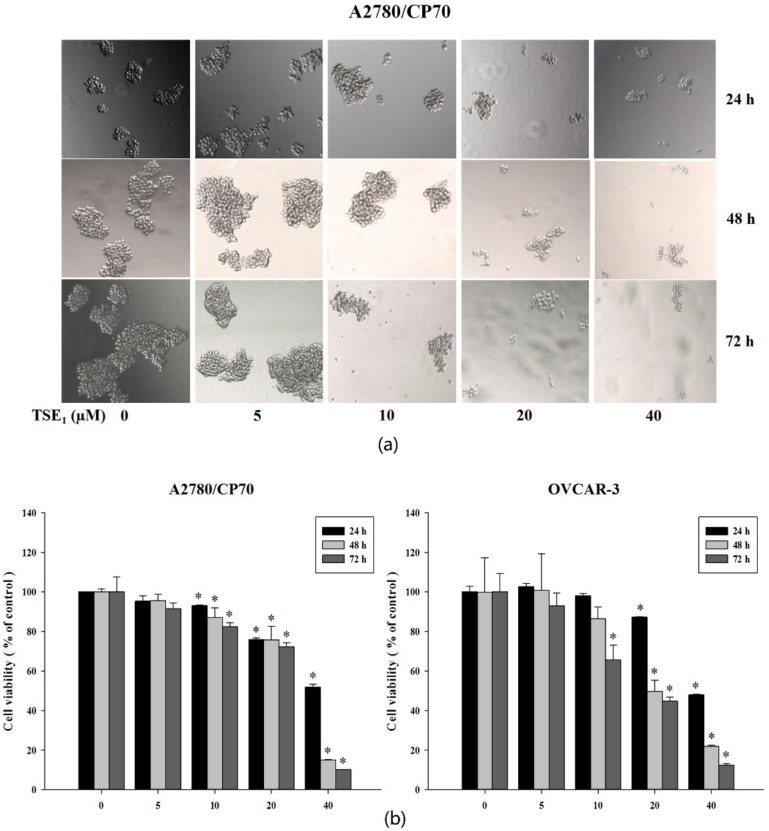
Effects of TSE_1_ on cell growth of sphere cells (3-weeks) from A2780/CP70 and OVCAR-3 cell lines. (**a**) cell morphology observation after treatment with various concentrations of TSE_1_ for 24, 48 and 72 h, respectively, via fluorescent microscopy (100×); (**b**) TSE_1_ inhibited cell viability of sphere cells with treatment 0 to 40 µM for 24, 48 and 72 h, respectively; cell viability was determined via MTS assay. Data was expressed as percent of control (without TSE_1_ treatment) and shown as mean ± SD (*n* = 5), * = *p* ≤ 0.05, significant difference compared to control at the same treatment time.

To investigate the anti-proliferation effect of TSE_1_ on both ALDH+ cells, we firstly observed the change of cell morphology under microscope after treatment of different concentrations of TSE_1_. As shown in Figure 5a, with the same treatment time, the number and size of suspended cells showed a tendency to decrease with increasing drug concentration. The same trend of action occurred for all three treatment times, showing a dose-dependent manner of the inhibitory effect of TSE_1_. While under the same drug concentration treatment, there was no obvious increasing trend in inhibitory effect within the prolonged action time. At low concentration of 5 µM, TSE_1_ had no effect on ALDH+ cells. Under the treatment of 10 µM TSE_1_, the size and number of suspended spheres were decreased to a certain extent. When the concentrations of TSE_1_ increased to 20 µM or 40 µM, its inhibitory effects on the formation size and number of suspended spheres were more pronounced.

Furthermore, the results of the cytotoxicity assay (MTS method) analysis were consistent with the observation of the growth tendency of the suspended spheres (Figure 5b). The viability of A2780/CP70 cells ranged from 95.31% to 51.92% at 24 h, from 95.62% to 15.06% at 48 h, and from 91.54% to 10.13% at 72 h. For the OVCAR-3 cell line, the viability was 102.56% to 47.99% at 24 h, 100.91% to 20.02% at 48 h, and 93.01% to 12.30% a 72 h. In addition, 5 µM TSE_1_ had no obvious effect on viability, even if the duration of action was extended to 72 h. With the increase of drug concentration, the inhibitory activity of TSE_1_ on ALDH+ cells was significantly increased, and the action time had a certain enhancement effect. When the concentration reached to 40 µM, prolonging the action time can significantly enhance the inhibitory effect of TSE_1_. The overall detection analysis was consistent with the observed cell morphology results. It was complemented that TSE_1_ could inhibit ALDH+ cells in a dose-dependent manner. The action time could enhance this inhibition to a certain extent, but it was not the main influence factor.

### 2.4. TSE_1_ Suppresses the Sphere Formation of Sphere Cells

**Figure 6:**
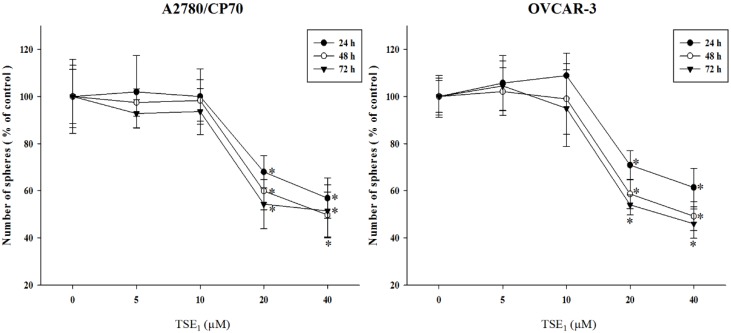
Effects of TSE_1_ on sphere formation of sphere cells (3-weeks) from A2780/CP70 and OVCAR-3 cell lines. TSE_1_ suppressed the sphere forming viability of sphere cells with treatment 0 to 40 µM for 24, 48 and 72 h, respectively. Data was expressed as percent of control (without TSE_1_ treatment) and shown as mean ± SD (*n* = 5), * = *p* ≤ 0.05, significant difference compared to control at the same treatment time.

In view of the MTS results, the ability of TSE_1_ to inhibit the proliferation of ALDH+ cells was achieved by inhibiting the size and number of the cell spheres. We further investigated whether TSE_1_ affects the ability of CSCs single cell to pellet. Figure 6 showed that TSE_1_ exhibited a similar trend for ALDH+ cells in two cell lines. At low concentrations of 5 μM and 10 μM TSE_1_, the number of single cells pelleted was not significantly different from that of the control group even when the action time was extended from 24 h to 72 h, indicating that low concentration had no effect on the single cell pelleting ability of ALDH+ cells, and the action time has no obvious effect on it. With the increase of TSE_1_ treatment concentration to 20 μM, the percentage of single cell pellets showed a significant decreasing trend under three action times, and the prolonged action time could enhance this effect to some extent, but there was no significant difference among them. When the concentration of TSE_1_ continued to increase to 40 μM, the inhibitory effect of TSE_1_ continued to increase, but the action time still had no significant effect. These results indicated that TSE_1_ can dose-dependently reduce the single cell pelleting ability of ALDH+ cells. This result was consistent with the MTS results.

## 3. Discussion

Accumulating evidence has proposed that CSCs play a key role in driving and sustaining tumor initiation, progression and malignant proliferation; CSCs were therefore believed to be the key target for prevention of tumor recurrence and metastasis [2,4]. Thus, target therapy for CSCs attracts more research interest and is considered a new therapeutic strategy against cancers.

In this study, our results showed that the ALDH+ cells in A2780/CP70 and OVCAR-3 tumor cells took up less than 2.0%, and could be significantly accumulated through the tumor sphere culture method. In addition, we found that ALDH+ cells possess higher sphere-forming ability and over-expression of stemness markers including CD44, Nanog, Oct-4, Bcl-2, Notch-1 and MMP-9 proteins. Clinical and experimental studies have indicated that over-expression of CD44 adhesion molecule contributes a poorer prognosis [20], and enhances tumorigenic and metastatic proclivity [21]. Higher levels of CD44 have been observed to play a major role in deregulated-splicing mediated ovarian cancer progression [22]. Pluripotent specific markers Nanog and Oct-4 were found to have induced the expression of each other [23,24]. Nanog was observed to drive the cancer stem cell tumorigenic potential and Oct-4 promotes invasion, metastasis and chemoresistance in ovarian cancer [6]. Higher protein levels of CD44, Nanog and Oct-4 observed in this study were inconsistent with the previous reports, indicating that the ALDH+ cells had some stemness properties. Moreover, antiapoptotic factor Bcl-2 and the migration related protein MMP-9 were significantly increased in ALDH+ cells compared with ALDH− tumor cells. Researchers have demonstrated that the Bcl-2 protein family plays an essential role in regulating intrinsic or mitochondrial apoptosis, and is strongly associated with drug resistance [25]. Over-expression of Bcl-2 protein has been observed in side population cells from high grade ovarian cancer [26]. In addition, MMP-9 plays an important role in the cell metastasis and invasion [27] and contributes to the ovarian tumor aggression and recurrence [22,28]. The observed over-expressed stemness markers could explain the significantly enhanced migration activity of ALDH+ cells from both the cell lines and the increased invasion ability of A2780/CP70 cell line. Overall, our data suggested that ALDH+ cells possessed some cancer stem cell-like properties including enhanced sphere formation, stronger migration ability and higher expression of stemness markers. 

The saponins have been found to have numerous pharmacological effects including anti-fungal [29], anti-viral [30], anti-inflammatory [31], anti-hyperlipidemic [32], immunomodulatory [33] and anti-tumor activities [17,19]. Among these, TSE_1_ was proved to have higher bioactivity than other saponin monomers present in tea seeds [17]. Researchers have shown that TSE_1_ expressed an inhibitory effect on ethanol-induced gastric mucosal lesions at a dose of 5.0 mg/kg [16]. TSE_1_ showed significant quinone reductase (QR) inducing activity at 4 µg/mL (3.25 µM) and could be used as an anti-tumor agent and a chemo-prevention agent of cancer [18]. Treating with a 10 µg/mL (8.13 µM) concentration of TSE_1_ completely inhibited tube formation in human umbilical vein endothelial cells, as well as TSE_1_ showing toxicity toward cancer cells and inhibiting in vivo growth of the tumor [17]. On the other hand, we carried out another study before this work, data showed the estimated half-maximal inhibitory concentration (IC_50_) of TSE_1_ for A2780/CP70 and OVCAR-3 tumor cells were 3.46 µM and 3.28 µM, respectively. Our results were consistent with those reported data. While the IC_50_ value of TSE_1_ for normal ovarian surface epithelial cell line IOSE-364 was 23.82 µM, TSE_1_ showed a much lower cytotoxic effect against normal ovarian epithelial cells. In addition, our published data showed that the IC_50_ values of cisplatin, which is the most effective and widely used chemotherapeutic agent, were 11.91 µg/mL (35.71 µM) for A2780/CP70 and 10.10 µg/mL (30.30 µM) for OVCAR-3, respectively [19]. All data taken together, we indicated that TSE_1_ exhibited higher inhibitory effects compared to cisplatin. However, few reports have been published to identify the beneficial biological activities of TSE_1_ on CSCs. In this study, we used ALDH+ cells with stemness properties as CSCs model to demonstrate the TSE_1_-mediated suppression on CSCs growth and sphere formation. Our results indicated that TSE_1_ had significant inhibitory activity against ALDH+ cells of A2780/CP70 and OVCAR-3 cell lines at concentrations of 20 and 40 μM, but exhibited little influence on both cell lines at lower concentrations of 5 or 10 μM. The present work indicated that higher TSE_1_ concentration was needed to inhibit ALDH+ cells with chemo-resistance capacity [6]. TSE_1_ decreased numbers of spheres and cell viability at higher concentrations. As reported, side population cells from high grade ovarian cancer showed high resistance to chemotherapy drugs, and high survival rate and high potential to form tumor spheres [26]. The inhibitory effects of TSE_1_ on cell proliferation and sphere-forming viability has certain significance to suppress cancer stem cells. However, while increasing TSE_1_ concentrations impacted the results, increasing the treatment times did not have significant effects. These results coincide with previous research showing that the cytotoxic effects of saponins on various cancer cell lines varied based on the type of cell line and on the concentration of saponins [34]. This research also added other evidence to previous research, which demonstrates the anti-proliferative, anti-tumor and cytotoxic effects of TSE_1_ [17,18]. These results suggest that TSE_1_ is a natural and potent drug candidate for ovarian cancer treatment. However, the molecular weight of tea saponin is 1230, which is much higher than cisplatin (the molecular weight is 300). Thus, the measurement of the bioavailability of TSE_1_ is very valuable for anti-tumor research and possible drug applications for the disease, as well as the molecular mechanism of TSE_1_-mediated inhibition of ALDH+ cells. 

## 4. Materials and Methods

### 4.1. General Experimental Procedures

**Figure 7:**
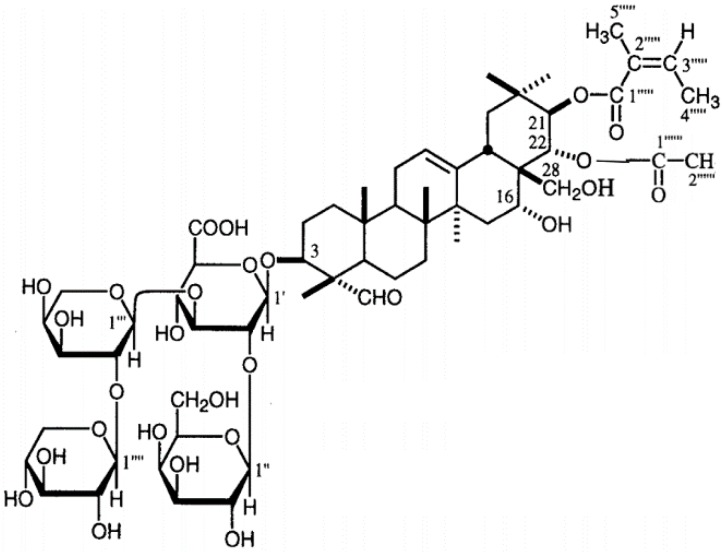
Chemical structure of theasaponin E_1_.

TSE_1_ (Figure 7) was purchased from Yingshili Biotechnology (Hangzhou, Zhejiang, China), and was dissolved in dimethyl sulfoxide (DMSO) at 100 mM and stored at −20 °C PLT, 6-well plates, 24-well plates, 96-well plates, Transwell chambers and Corning Matrigel Basement Membrane Matrix High Concentration were from Corning (Corning, NY, USA). Aqueous One Solution Cell Proliferation assay (MTS) was purchased from Promega Corporation (Madison, WI, USA). RPMI-1640 medium was purchased from Sigma-Aldrich (St. Louis, MO, USA). Fetal bovine serum (FBS) was from Invitrogen (Grand Island, NY, USA). ALDEFLUOR Stem Cell Identification Kit, MammoCult Medium Human Kit, Hydrocortisone Stock Solution, 0.2% Heparin Solution and HBSS were obtained from Stem Cell Technologies (Vancouver, BC, Canada). Primary antibodies for Oct-4, CD44, Nanog, Bcl-2, Notch-1, MMP-9 and horseradish peroxidase-conjugated secondary antibody were purchased from Cell Signaling Technology, Inc. (Danvers, MA, USA). Antibody for GAPDH was obtained from Santa Cruz Biotechnology, Inc. (Dallas, TX, USA).

### 4.2. Cell Culture

Human platinum-resistant ovarian cancer cell lines A2780/CP70 (p53 wild-type) and OVCAR-3 (p53 mutant) regarded as a gift were kindly provided by Dr. Jiang at West Virginia University. All cells were cultured in RPMI-1640 medium supplemented with 10% FBS in a humidified incubator with 5% CO_2_ at 37 °C. Then, cells were subsequently treated through tumor sphere culture for a certain time adjusted by each experiment.

### 4.3. Tumor Sphere Culture

This progress was performed under serum-free medium and ultra-low adherent conditions according to the manufacturer’s instructions. Briefly, both A2780/CP70 and OVCAR-3 ovarian cancer cells (tumor cells) were collected when 70% density was achieved and then suspended into the complete mammocul^TM^ medium (complete medium below) supplemented with 4 μg/mL (final concentration) 0.2% Heparin Sodium Salt and 0.48 μg/mL (final concentration) Hydrocortisone Stock Solution. Cells were seeded at a density of 6,000 cells/well in 6-well ultra-low attachment plates, then cultured for 7 days at 37 °C in a humidified incubator with 5% CO_2_, and visualized under a light microscope every day. The spheres were harvested after 7 days of culture for subsequent tumor sphere culture using the same method described, and subsequent assays.

### 4.4. ALDEFLUORTM Kit ALDH Analysis

The population of ALDH in tumor and sphere cells (1-week, 2-weeks and 3-weeks) were analyzed by a ALDEFLUOR Stem Cell Identification Kit according to the manufacturer’s instructions. All cells were harvested from dishes with Trypsin-EDTA, then suspended using ALDEFLUOR^TM^ Assay Buffer to prepare samples with a density of 10^6^ cells/mL. Then, 1 mL sample was added into a test tube with 5 μL activated ALDEFLUOR^TM^ Reagent and mixed rapidly. Next, 0.5 mL mixture was transferred to the control tube with an added 5 μL ALDEFLUOR^TM^ DEAB Reagent prior. All test and control samples were mixed quickly, and then cultured for 45 min at 37 °C in a humidified atmosphere. Then, all samples were centrifuged at 250× *g* for 5 min at 4 °C and suspended in 0.5 mL ALDEFLUOR^TM^ assay buffer. All samples were stored on ice before detection. Finally, all samples were filtered through a 40 μm cell trainer to obtain single suspension cells for analysis on a FACSAria III Flow cytometer (BD).

### 4.5. Sphere Formation Assay

Tumor and sphere cells (1-week, 2-weeks and 3-weeks) were harvested, then lightly centrifuged at 250× *g* for 5 min at room temperature, and resuspended using complete medium. All cells were plated at a density of 100 cells/well in 96-well ultra-low adherent plates, and cultured for a subsequent 7 days in a humidified incubator containing 5% CO_2_ at 37 °C. The cells were visualized and the number of spheres (>50 μm) formed were counted [27] under a light microscope every day. All data were collected from six independent trials to determine the inhibition effect of sphere formation by TSE_1_. Sphere cells (3-weeks) were harvested and seeded under the same condition, and cultured for 24 h. Then, cells were treated with various concentrations of TSE_1_ (0 to 40 μg/mL) for 24, 48 and 72 h. The control group received only an equal amount of complete medium. Then, the number of spheres was counted as above. All data were collected from five independent experiments

### 4.6. Western Blot Analysis

Ovarian cancer cells were seeded and incubated overnight until 70% density was achieved. Sphere cells (3-weeks) were collected after 7 days of culture. All cells were centrifuged at 350× *g* for 5 min at 4 °C to remove the old medium. After a double wash with cold PBS, cells were lysed in 50 μL M-PER Mammalian Protein Extraction Reagent supplemented with 1 μL Halt^TM^ Protease and Phosphatas Inhibitor. Total protein levels were determined by a BCA Protein Assay Kit. The cell lysates were separated by SDS-PAGE and blotted onto a nitrocellulose membrane with a Mini-Protean 3 System (Bio-Rad, Hercules, CA, USA). Membranes were blocked with 5% non-fat milk (dissolved in Tris-buffer saline supplemented with 0.1% Tween 20 (TBST)) for 1 h at room temperature. Then, the membranes were incubated with specific primary antibodies and secondary antibody dilutions. After washing with TBST in triplicate, the antigen-antibody complex was visualized with ECL Kit. Protein bands were quantified with NIH Image J software (version, NIH, Bethesda, MD, USA), and normalized by corresponding GAPDH bands for analysis.

### 4.7. Transwell Migration and Invasion Assays

In addition, 50 µL 1/8 dilution of matrigel was seeded into upper Transwell chambers and solidified in a humidified incubator at 37 °C for 30 min to firm membrane for subsequent invasion assay. Both A2780/CP70 and OVCAR-3 tumor and sphere cells (3-weeks) in suspension serum-free medium were plated into the upper chambers at 2 × 10^5^ cells/well for invasion assay, and migration assay without matrigel membrane. A complete medium containing 20% FBS was added to the lower chamber and incubated for another 24 h. The medium was wiped in the upper chambers and washed with PBS twice at room temperature. A cotton swab was used to gently wipe out the residual solution and then crystal violet staining cells were passed through the membrane and attached to the lower layer of the membrane for at least 15 min after formaldehyde fixing cells for 15 min. Graphics of cells that migrated or invaded were taken using wide-field microscopy.

### 4.8. Cell Viability

Cell growth inhibition was determined by using MTS assay as described as before [19]. Sphere cells (3-weeks) were seeded into 96-well ultra-low adherent plates at a density of 2000 cells/well in complete medium for 24 h. Then, cells were treated with various concentrations of TSE_1_ (0 to 40 μg/mL) for 24, 48 and 72 h at 37 °C in a humidified 5% CO_2_ incubator. The control group received only an equal amount of complete medium. Then, morphologic observation for spheres was carried out under a light microscope before cell viability analysis. For MTS assay, 100 μL of MTS was added to each well and incubated in the dark at 37 °C for 1 h. The absorbance was measured at 490 nm with a microplate reader. Cell viability was expressed as the percentage compared to the untreated group. All data was collected from five independent experiments. 

### 4.9. Statistical Analysis

In this study, the experiments were performed at least three times. Average values of replicates were collected and analyzed by one-way analysis of variance (ANOVA) in SPSS 23.0 (Manufacturer, City, US State abbrev. if applicable, Country). Significant differences between individual treatments and control were evaluated using Duncan’s test. * = *p* ≤ 0.05 was considered statistically significant.

## 5. Conclusions

In this work, the data showed that a rare population of CSCs (less than 2.0%) existed in A2780/cp70 and OVCAR-3 cell lines and CSCs were noticeably accumulated through serum-free spheroid formation assay. Most importantly, we demonstrated that ALDH+ cells performed stronger single cell sphering and migration ability, and demonstrated over-expression of stemness marker proteins CD44, Nanog and Oct-4, and cancer stem cell-associated proteins Bcl-2 and MMP-9, thus indicating some biological characteristics of cancer stem cells, which can be considered as ovarian cancer stem-like cells. In addition, we indicated that TSE_1_ dose-dependently inhibited the proliferation and single cell sphere formation ability, though it was not significantly changed by action time. This study provided new evidence for the anti-tumor activity of TSE_1_. We suggest that TSE_1_ has potential application for use in chemotherapy options to treat advanced ovarian cancer efficiency in the future. More research should be aimed at the underlying molecular mechanisms of TSE_1_-mediated suppression ability on ALDH+ cells for better utility of natural compound of TSE_1_ in tumor therapy.

## Abbreviations

ALDH	Aldehyde dehydrogenase
CSCs	Cancer stem cells
TSE_1_	Theasaponins E_1_
